# Supplementary material for: Mitochondrial HSP70 Chaperone System—The Influence of Post-Translational Modifications and Involvement in Human Diseases
Source: Int J Mol Sci. 2021 Jul 28;22(15):8077. doi: 10.3390/ijms22158077 (PMC8347752; doi:10.3390/ijms22158077)
Supplement: Supplementary file 1 [file ijms-22-08077-s001.zip › ijms-1301111-supplementary.pdf]

Mitochondrial HSP70 Chaperone System – the Influence of Post-translational Modifications and Involvement in Human Diseases

Havalová H., *et al.*

Supplementary Table S1

Table S1. The overview of mortalin post-translational modifications and their involvement in human diseases, which appear in the PhosphositePlus® Database. Only modifications identified by at least five separate proteomic studies are listed.

Modification	Position	Localization in domain structure	Diseases	Tissue
**Phosphorylation**	S3	MTS	breast cancer [1]	MCF10A1 (EP) [1], MCF10AT1K (EP) [1], MCF10CA1a (EP) [1], MCF10CA1h (EP) [1]
	S5	MTS	breast cancer [1]	MCF10A1 (EP) [1], MCF10AT1K (EP) [1], MCF10CA1a (EP) [1], MCF10CA1h (EP) [1]
	T22	MTS	breast cancer [1]	MCF10A1 (EP) [1], MCF10AT1K (EP) [1], MCF10CA1a (EP) [1], MCF10CA1h (EP) [1]
	S29	MTS	breast cancer [2]	breast [2], Jurkat (TL) [3]
	Y46	MTS	lung cancer [4]	CL1-5 (PL) [4]
	T87	NBD	lung cancer [5]	DMS153 (PL), K562 (ERT) [6], lung [5], NCI-H1437 (PL), NCI-H2073 (PL), NCI-H209 (PL), NCI-H838 (PL)
	Y128	NBD	gastric cancer, leukemia, chronic myelogenous leukemia, lung cancer	A549 (PL), Cal-12T (PL), DMS153 (PL), HCC15 (PL), HCC44 (PL), HCC78 (PL), K562 (ERT), LOU-NH91 (SQ), lung, MKN-45 (GAS), NCI-H1299 (PL), NCI-H1355 (PL), NCI-H1437 (PL), NCI-H1666 (PL), NCI-H1703 (SQ), NCI-H1734 (PL), NCI-H1781 (PL), NCI-H1792 (PL), NCI-H1944 (PL), NCI-H2073 (PL), NCI-H209 (PL), NCI-H2228 (PL), NCI-H23 (PL), NCI-H2342 (PL), NCI-H2405 (PL), NCI-H3122 (PL), NCI-H3255 (PL), NCI-H358 (PL), NCI-H441 (PL), NCI-H460 (PL), NCI-H661 (PL), NCI-H838 (PL)

S148	NBD	breast cancer [2,7]	breast [2], BT-549 (BC) [7] , MDA-MB-231 (BC) [7]
S162	NBD	lung cancer [8]	Jurkat (TL) [3], K562 (ERT) [6], PC9 (PL) [8], PC9-IR (PL) [8]
S164	NBD	breast cancer [2], lung cancer [8]	breast [2], Jurkat (TL), PC9-IR (PL) [8]
S200	NBD	breast cancer [2]	breast [2], HEK293T (EP) [9], HeLa (CER) [10,11], Jurkat (TL)
T271	NBD	lung cancer	A549 (PL), Cal-12T (PL), Calu-3 (PL), DMS153 (PL), DMS53 (PL), HCC15 (PL), HCC44 (PL), HCC78 (PL), HCC827 (PL), LOU-NH91 (SQ), lung, NCI-H1299 (PL), NCI-H1355 (PL), NCI-H1437 (PL), NCI-H1650 (PL), NCI-H1666 (PL), NCI-H1703 (SQ), NCI-H1734 (PL), NCI-H1781 (PL), NCI-H1792 (PL), NCI-H1944 (PL), NCI-H1975 (PL), NCI-H2073 (PL), NCI-H209 (PL), NCI-H2106 (PL), NCI-H2228 (PL), NCI-H23 (PL), NCI-H2342 (PL), NCI-H2405 (PL), NCI-H3122 (PL), NCI-H358 (PL), NCI-H441 (PL), NCI-H446 (PL), NCI-H460 (PL), NCI-H526 (PL), NCI-H661 (PL), NCI-H69 (PL), NCI-H82 (PL), NCI-H838 (PL)
S378	NBD	erythroid leukemia [12], acute myeloblastic leukemia [12], lung cancer, B cell lymphoma [12], non-Hodgkin's lymphoma [12], multiple myeloma [12]	AML-193 (MN) [12], CMK (MGK) [12], CTS (MYE) [12], DOHH2 (BL, precursor) [12], HEL (ERT) [12], HeLa (CER) [10], Kasumi-1 (MYE) [12], KG-1 (MYE) [12], lung , MV4-11 (MP) [12], OPM-2 (PC) [12], P31/FUJ (ERT) [12], RL (BL, precursor) [12], RPMI-8266 (PC) [12], SU-DHL-6 (BL) [12], U266 (PC) [12], Vero E6-S (EP, KD) [13]
S408	NBD	breast cancer [2]	breast [2], HeLa (CER) [11], K562 (ERT) [6]
Y568	SBD	cervical cancer [14], gastric cancer, leukemia, chronic myelogenous leukemia, liver	DMS153 (PL), HCC15 (PL), HCC78 (PL), HeLa S3 (CER) [14], K562 (ERT), liver, LOU-NH91 (SQ), lung, NCI-H1437 (PL), NCI-H1703 (SQ), NCI-H1781 (PL), NCI-H2073 (PL), NCI-H209 (PL),

			cancer, hepatocellular carcinoma, lung cancer	NCI-H2228 (PL), NCI-H2342 (PL), NCI-H3122 (PL), NCI-H661 (PL), NCI-H838 (PL), stomach
	S627	SBD	breast cancer [2], melanoma skin cancer [15]	breast [2], MCF-7 (BC), WM239A (MEL) [15]
**Mono-methylation**	R513	SBD	gastric cancer, lung cancer	293 (EP) [16], A549 (PL), Cal-12T (PL), Calu-3 (PL), DMS153 (PL), DMS53 (PL), HCC15 (PL), HCC44 (PL), HCC78 (PL), HCC827 (PL), HCT116 (INT) [17], Jurkat (TL) [18], LOU-NH91 (SQ), lung, MKN-45 (GAS), NCI-H1299 (PL), NCI-H1355 (PL), NCI-H1437 (PL), NCI-H1650 (PL), NCI-H1666 (PL), NCI-H1703 (SQ), NCI-H1734 (PL), NCI-H1781 (PL), NCI-H1792 (PL), NCI-H1944 (PL), NCI-H1975 (PL), NCI-H2073 (PL), NCI-H209 (PL), NCI-H2106 (PL), NCI-H2228 (PL), NCI-H23 (PL), NCI-H2342 (PL), NCI-H2405 (PL), NCI-H3122 (PL), NCI-H3255 (PL), NCI-H358 (PL), NCI-H441 (PL), NCI-H446 (PL), NCI-H460 (PL), NCI-H526 (PL), NCI-H661 (PL), NCI-H69 (PL), NCI-H82 (PL), NCI-H838 (PL), TL [18]
**Acetylation**	K121	NBD	lung cancer, B cell lymphoma	NCI-H2228 (PL), RC-K8 (BL)
	K135	NBD	colorectal cancer, leukemia, chronic myelogenous leukemia, liver cancer, hepatocellular carcinoma, lung cancer [19]	A549 (PL) [19,20], HCT116 (INT), HeLa (CER) [21], HepG2 (HP), Jurkat (TL) [3,20], K562 (ERT), liver [22], MV4-11 (MP) [20], RC-K8 (BL)
	K138	NBD	leukemia, chronic myelogenous leukemia, lung cancer [19]	A549 (PL) [19,20], HeLa (CER) [21], Jurkat (TL) [3,20], K562 (ERT)
	K159	NBD	colorectal cancer, gastric cancer, leukemia, chronic	HCT116 (INT), HeLa (CER) [21], K562 (ERT), MKN-45 (gastric),

		myelogenous leukemia, lung cancer	NCI-H1703 (SQ), NCI-H2228 (PL)
K206	NBD	not found	HeLa (CER) [21], Jurkat (TL) [3]
K288	NBD	colorectal cancer, leukemia, chronic myelogenous leukemia, liver cancer, lung cancer [19], ventricular tachycardia	A549 (PL) [19,20], cardiac-heart, HCT116 (INT), HCT8/S11 (INT), HT-29 (INT), Jurkat (TL) [3,20], K562 (ERT), liver [22], MV4-11 (MP) [20], NCI-H2228 (PL), NCI-H716 (INT), OCI-ly3 (BL), RC-K8 (BL), SEM (BL), SNU-C2B (INT), SW480 (INT), SW620 (INT), UOK-101 (REN)
K291	NBD	ventricular tachycardia	cardiac-heart
K300	NBD	colorectal cancer, leukemia, chronic myelogenous leukemia, liver cancer, hepatocellular carcinoma, lung cancer [19]	A549 (PL) [19,20], HCT116 (INT), HeLa (CER) [21], HepG2 (HP), Jurkat (TL) [3], K562 (ERT)
K345	NBD	gastric cancer, lung cancer [19]	A549 (PL) [19], DMS79 (PL), Jurkat (TL) [20], lung, MKN-45 (GAS), NCI-H128 (PL), NCI-H1299 (PL), NCI-H1417 (PL), NCI-H1703 (SQ), NCI-H1734 (PL), NCI-H1944 (PL), NCI-H209 (PL), NCI-H358 (PL), NCI-H460 (PL), NCI-H524 (PL)
K567	SBD	colorectal cancer, leukemia, chronic myelogenous leukemia, lung cancer	A549 (PL) [20], Cal-12T (PL), Calu-3 (PL), DMS153 (PL), DMS53 (PL), DMS79 (PL), HCC15 (PL), HCC44 (PL), HCC827 (PL), HCT116 (INT), HeLa (CER) [21], Jurkat (TL) [3,20], K562 (ERT), LOU-NH91 (SQ), lung, MV4-11 (MP) [20], NCI-H128 (PL), NCI-H1299 (PL), NCI-H1355 (PL), NCI-H1417 (PL), NCI-H1437 (PL), NCI-H1650 (PL), NCI-H1666 (PL), NCI-H1703 (SQ), NCI-H1734 (PL), NCI-H1792 (PL), NCI-H1944 (PL), NCI-H1975 (PL), NCI-H2073 (PL), NCI-H209 (PL),

				NCI-H2106 (PL), NCI-H2228 (PL), NCI-H23 (PL), NCI-H2342 (PL), NCI-H2405 (PL), NCI-H3255 (PL), NCI-H358 (PL), NCI-H441 (PL), NCI-H446 (PL), NCI-H460 (PL), NCI-H524 (PL), NCI-H526 (PL), NCI-H69 (PL), NCI-H82 (PL), NCI-H838 (PL)
	K600	SBD	liver cancer, lung cancer [19]	A549 (PL) [19], HeLa (CER) [21], Jurkat (TL) [3], K562 (ERT), liver
	K612	SBD	colorectal cancer, leukemia, chronic myelogenous leukemia, lung cancer [19]	A549 (PL) [19], HCT116 (INT), Jurkat (TL) [3], K562 (ERT)
	K646	SBD	gastric cancer, lung cancer	A549 (PL) [20], lung, MKN-45 (GAS), NCI-H1299 (PL), NCI-H1703 (SQ), NCI-H1734 (PL), NCI-H1944 (PL), NCI-H358 (PL), NCI-H460 (PL), RC-K8 (BL)
	K675	SBD	leukemia, chronic myelogenous leukemia	K562 (ERT)
	K678	SBD	kidney cancer, leukemia, chronic myelogenous leukemia	K562 (ERT)
**Ubiquitylation**	K121	NBD	multiple myeloma	HEp-2 (SQ) [23], Jurkat (TL) [3,24], RPMI-8266 (PC)

Abbreviations

BL	B lymphocyte
BC	breast cell
CER	cervical
EP	epithelial
ERT	erythroid
GAS	gastric
HP	hepatic
INT	intestinal
KD	kidney
MP	macrophage
MGK	megakaryoblast
MEL	melanocyte
MN	monocyte
MYE	myeloid
PC	plasma cell

PL	pulmonary
REN	renal
SQ	squamous
TL	T lymphocyte

References:

1. Chen, Y.; Choong, L.Y.; Lin, Q.; Philp, R.; Wong, C.H.; Ang, B.K.; Tan, Y.L.; Loh, M.C.; Hew, C.L.; Shah, N.*, et al.* Differential expression of novel tyrosine kinase substrates during breast cancer development. *Mol Cell Proteomics* **2007**, *6*, 2072-2087.
2. Mertins, P.; Mani, D.R.; Ruggles, K.V.; Gillette, M.A.; Clauser, K.R.; Wang, P.; Wang, X.; Qiao, J.W.; Cao, S.; Petralia, F.*, et al.* Proteogenomics connects somatic mutations to signalling in breast cancer. *Nature* **2016**, *534*, 55-62.
3. Mertins, P.; Qiao, J.W.; Patel, J.; Udeshi, N.D.; Clauser, K.R.; Mani, D.R.; Burgess, M.W.; Gillette, M.A.; Jaffe, J.D.; Carr, S.A. Integrated proteomic analysis of post-translational modifications by serial enrichment. *Nat Methods* **2013**, *10*, 634-637.
4. Wu, H.Y.; Tseng, V.S.; Chen, L.C.; Chang, Y.C.; Ping, P.; Liao, C.C.; Tsay, Y.G.; Yu, J.S.; Liao, P.C. Combining alkaline phosphatase treatment and hybrid linear ion trap/orbitrap high mass accuracy liquid chromatography-mass spectrometry data for the efficient and confident identification of protein phosphorylation. *Anal Chem* **2009**, *81*, 7778-7787.
5. Schweppe, D.K.; Rigas, J.R.; Gerber, S.A. Quantitative phosphoproteomic profiling of human non-small cell lung cancer tumors. *J Proteomics* **2013**, *91*, 286-296.
6. Zhou, H.; Di Palma, S.; Preisinger, C.; Peng, M.; Polat, A.N.; Heck, A.J.; Mohammed, S. Toward a comprehensive characterization of a human cancer cell phosphoproteome. *J Proteome Res* **2013**, *12*, 260-271.
7. Klammer, M.; Kaminski, M.; Zedler, A.; Oppermann, F.; Blencke, S.; Marx, S.; Muller, S.; Tebbe, A.; Godl, K.; Schaab, C. Phosphosignature predicts dasatinib response in non-small cell lung cancer. *Mol Cell Proteomics* **2012**, *11*, 651-668.
8. Tsai, C.F.; Wang, Y.T.; Yen, H.Y.; Tsou, C.C.; Ku, W.C.; Lin, P.Y.; Chen, H.Y.; Nesvizhskii, A.I.; Ishihama, Y.; Chen, Y.J. Large-scale determination of absolute phosphorylation stoichiometries in human cells by motif-targeting quantitative proteomics. *Nat Commun* **2015**, *6*, 6622.
9. Matsuoka, S.; Ballif, B.A.; Smogorzewska, A.; McDonald, E.R., 3rd; Hurov, K.E.; Luo, J.; Bakalarski, C.E.; Zhao, Z.; Solimini, N.; Lerenthal, Y.*, et al.* Atm and atr substrate analysis reveals extensive protein networks responsive to DNA damage. *Science* **2007**, *316*, 1160-1166.
10. Kettenbach, A.N.; Schweppe, D.K.; Faherty, B.K.; Pechenick, D.; Pletnev, A.A.; Gerber, S.A. Quantitative phosphoproteomics identifies substrates and functional modules of aurora and polo-like kinase activities in mitotic cells. *Sci Signal* **2011**, *4*, rs5.
11. Sharma, K.; D'Souza, R.C.; Tyanova, S.; Schaab, C.; Wisniewski, J.R.; Cox, J.; Mann, M. Ultradeep human phosphoproteome reveals a distinct regulatory nature of tyr and ser/thr-based signaling. *Cell Rep* **2014**, *8*, 1583-1594.
12. Casado, P.; Alcolea, M.P.; Iorio, F.; Rodriguez-Prados, J.C.; Vanhaesebroeck, B.; Saez-Rodriguez, J.; Joel, S.; Cutillas, P.R. Phosphoproteomics data classify hematological cancer cell lines according to tumor type and sensitivity to kinase inhibitors. *Genome Biol* **2013**, *14*, R37.
13. Bouhaddou, M.; Memon, D.; Meyer, B.; White, K.M.; Rezelj, V.V.; Correa Marrero, M.; Polacco, B.J.; Melnyk, J.E.; Ulferts, S.; Kaake, R.M.*, et al.* The global phosphorylation landscape of sars-cov-2 infection. *Cell* **2020**, *182*, 685-712 e619.
14. Olsen, J.V.; Vermeulen, M.; Santamaria, A.; Kumar, C.; Miller, M.L.; Jensen, L.J.; Gnad, F.; Cox, J.; Jensen, T.S.; Nigg, E.A.*, et al.* Quantitative phosphoproteomics reveals widespread full phosphorylation site occupancy during mitosis. *Sci Signal* **2010**, *3*, ra3.
15. Stuart, S.A.; Houel, S.; Lee, T.; Wang, N.; Old, W.M.; Ahn, N.G. A phosphoproteomic comparison of b-rafv600e and mkk1/2 inhibitors in melanoma cells. *Mol Cell Proteomics* **2015**, *14*, 1599-1615.
16. Larsen, S.C.; Sylvestersen, K.B.; Mund, A.; Lyon, D.; Mullari, M.; Madsen, M.V.; Daniel, J.A.; Jensen, L.J.; Nielsen, M.L. Proteome-wide analysis of arginine monomethylation reveals widespread occurrence in human cells. *Sci Signal* **2016**, *9*, rs9.
17. Guo, A.; Gu, H.; Zhou, J.; Mulhern, D.; Wang, Y.; Lee, K.A.; Yang, V.; Aguiar, M.; Kornhauser, J.; Jia, X.*, et al.* Immunoaffinity enrichment and mass spectrometry analysis of protein methylation. *Mol Cell Proteomics* **2014**, *13*, 372-387.
18. Geoghegan, V.; Guo, A.; Trudgian, D.; Thomas, B.; Acuto, O. Comprehensive identification of arginine methylation in primary t cells reveals regulatory roles in cell signalling. *Nat Commun* **2015**, *6*, 6758.

19. Wu, Q.; Cheng, Z.; Zhu, J.; Xu, W.; Peng, X.; Chen, C.; Li, W.; Wang, F.; Cao, L.; Yi, X.*, et al.* Suberoylanilide hydroxamic acid treatment reveals crosstalks among proteome, ubiquitylome and acetylome in non-small cell lung cancer a549 cell line. *Sci Rep* **2015**, *5*, 9520.
20. Choudhary, C.; Kumar, C.; Gnad, F.; Nielsen, M.L.; Rehman, M.; Walther, T.C.; Olsen, J.V.; Mann, M. Lysine acetylation targets protein complexes and co-regulates major cellular functions. *Science* **2009**, *325*, 834-840.
21. Weinert, B.T.; Scholz, C.; Wagner, S.A.; Iesmantavicius, V.; Su, D.; Daniel, J.A.; Choudhary, C. Lysine succinylation is a frequently occurring modification in prokaryotes and eukaryotes and extensively overlaps with acetylation. *Cell Rep* **2013**, *4*, 842-851.
22. Zhao, S.; Xu, W.; Jiang, W.; Yu, W.; Lin, Y.; Zhang, T.; Yao, J.; Zhou, L.; Zeng, Y.; Li, H.*, et al.* Regulation of cellular metabolism by protein lysine acetylation. *Science* **2010**, *327*, 1000-1004.
23. Akimov, V.; Barrio-Hernandez, I.; Hansen, S.V.F.; Hallenborg, P.; Pedersen, A.K.; Bekker-Jensen, D.B.; Puglia, M.; Christensen, S.D.K.; Vanselow, J.T.; Nielsen, M.M.*, et al.* Ubisite approach for comprehensive mapping of lysine and n-terminal ubiquitination sites. *Nat Struct Mol Biol* **2018**, *25*, 631-640.
24. Udeshi, N.D.; Svinkina, T.; Mertins, P.; Kuhn, E.; Mani, D.R.; Qiao, J.W.; Carr, S.A. Refined preparation and use of anti-diglycine remnant (k-epsilon-gg) antibody enables routine quantification of 10,000s of ubiquitination sites in single proteomics experiments. *Mol Cell Proteomics* **2013**, *12*, 825-831.
